# Beyond trust: Implementation of urinary cotinine testing in lung transplant candidate evaluation

**DOI:** 10.1016/j.jhlto.2026.100709

**Published:** 2026-10-16

**Authors:** R. van Pel, L.M. Bek, K.D. von der Thüsen, A.J. Muntinga, M. den Blanken, J.G.J.V. Aerts, B.J. Mathot, F. Hofman, L. Seghers, K.I.M. Looman, M.E. Hellemons

**Affiliations:** a Department of Respiratory Medicine, Erasmus MC, University Medical Center Rotterdam, Rotterdam, The Netherlands; b Erasmus MC Transplantation Institute, Department of Respiratory Medicine, Erasmus University Medical Center, Rotterdam, The Netherlands; c Department of Cardiothoracic Surgery, Erasmus MC, University Medical Center Rotterdam, Rotterdam, The Netherlands

**Keywords:** Lung transplantation, Cotinine, Nicotine addiction, Exposure, Implementation routine testing

## Abstract

**Background:**

Nicotine cessation is a prerequisite for lung transplantation (LTx). Although urinary cotinine testing (COT) objectively verifies abstinence, its use in LTx referral workup remains understudied. This study examined how COT is implemented during LTx referral and factors associated with test ordering. Additionally, we assessed the prevalence of active nicotine use at referral, factors linked to ongoing exposure, and relapse into active smoking post-LTx.

**Methods:**

We conducted a retrospective single-center cohort study of all adults referred for LTx evaluation at our academic center between January 1, 2012, and December 31, 2025, using routinely collected clinical data, including COT. Baseline characteristics, COT ordering patterns, and assessment and post-transplant outcomes were analyzed.

**Results:**

Of the 1,326 patients referred for LTx evaluation, 574 (43%) underwent COT. Tested patients were older and more often had COPD, a history of smoking, and smoking household members, whereas patients with non-modifiable contraindications, those deemed too early for LTx, or those who declined LTx were less likely to be tested. Among tested patients, cotinine positivity (110/574 [19%]) was associated with younger age, COPD, smoking history, and shorter reported nicotine cessation. Cumulative smoking resumption was 1.0% at 1 year and 4.2% at 5 years post-LTx.

**Conclusion:**

COT during LTx evaluation is applied inconsistently, driven by patient characteristics and selective clinical judgment. While a considerable proportion of tested candidates showed positive nicotine exposure at referral, post-LTx smoking resumption rates were low. These findings support further evaluation of a structured, risk-based COT approach to detect nicotine use early, optimize candidate selection, preclude inappropriate donor organ allocation, and reach the best LTx outcomes.

## Background

Lung transplantation (LTx) is a lifesaving yet highly impactful and resource-intensive treatment for patients with end-stage lung disease.^1^ Because most LTx candidates have a history of tobacco use, underlying the predominance of chronic obstructive pulmonary disease (COPD) and interstitial lung disease (ILD) as major indications for LTx, smoking relapse after transplantation remains a clinically relevant concern. Despite comprehensive pre-transplant counseling and rigorous evaluation procedures, 12% to 19% of LTx recipients resume smoking post-LTx.^2^, ^3^, ^4^ Persistent nicotine addiction represents an absolute contraindication to LTx, not only for ethical considerations related to organ stewardship, but also because ongoing nicotine use after LTx is strongly associated with adverse post-transplant outcomes, including impaired pulmonary function or chronic lung allograft dysfunction, cardiovascular events, reduced renal function, and an increased incidence of malignancies.^2^, ^5^, ^6^, ^7^ Given the persistent mismatch between donor lung supply and demand, rigorous candidate selection is critical to ensure that LTx yields maximal long-term benefit.^1^, ^8^ Accordingly, the ISHLT consensus document on LTx candidate selection requires candidates to demonstrate abstinence from all tobacco and nicotine products prior to transplant.^9^ However, smoking status is frequently assessed through self-report, despite evidence that a substantial proportion of candidates falsely report nicotine abstinence.^10^, ^11^ Concerns about delayed listing or exclusion from transplant eligibility may drive underreporting, resulting in an underestimation of active smoking and potentially inappropriate organ allocation with consequently poorer post-transplant outcomes.^12^ Therefore, objective biochemical verification of abstinence is preferred.^9^ Urinary cotinine is a widely used and stable biomarker of nicotine exposure due to its relatively long half-life (~20 hours).^13^ As such, this provides sensitive detection of active smoking, nicotine replacement therapy, and in the lower range passive smoking exposure.^14^ Although urinary cotinine testing (COT) is well established for monitoring post-transplant smoking relapse, far less is known about its use during the pre-transplant evaluation phase. Clinical practice appears to vary considerably across physicians and centers, influenced by local workflows, patient characteristics, and the absence of standardized implementation protocols.^15^ Existing studies have largely focused on the prevalence of nicotine use pre- and post-LTx,^2^, ^3^, ^4^, ^11^ providing almost no insight into how COT is implemented in daily practice, how consistently tests are ordered, or which factors drive variation in implementation. As a result, it remains unclear whether current screening practices adequately identify all candidates with ongoing nicotine exposure, despite the clinical and ethical importance of doing so. To address this, we aimed to examine how routinely and consistently COT is applied during lung transplant assessment and identify factors associated with test ordering. In addition, we aimed to assess the prevalence of a positive cotinine test result at the time of referral and to identify demographic and clinical factors associated with ongoing nicotine exposure. Last, we assessed the relapse rate into active smoking after LTx.

## Methods

### Study design

We conducted a retrospective single-center cohort study including all adult patients (≥18 years) referred to our LTx center for LTx assessment from January 1, 2012, to December 31, 2025. Data lock was July 1, 2026, and follow-up for smoking resumption after Ltx was censored on December 31, 2025. At the initial visit, patients underwent a standardized clinical evaluation of eligibility, including assessment of absolute contraindications and lung disease severity, and received information about the LTx procedure and its implications. COT became available during the study period after 2012 and was subsequently intended for routine use at the initial visit. In practice, COT was performed at the discretion of the treating physician, depending on the referral context and clinical judgment. After transplantation, COT is performed routinely every 6 to 12 months in all patients under follow-up. In addition to self-report, this surveillance strategy enables the detection of relapse of tobacco use. Routinely collected data included baseline characteristics and data regarding COT ordering and post-LTx follow-up. The Medical Ethics Committee of the Erasmus Medical Center approved the study (MEC-2025–0632) and waived the requirement for informed consent.

### Baseline characteristics

Demographics included patients’ age, sex, referral period, underlying diseases, smoking status, pack-years, time since nicotine cessation, and the presence of smoking household members. The underlying diseases were categorized as COPD (including alpha-1-antitrypsin deficiency), ILD, cystic fibrosis (CF)/non-CF bronchiectasis (BE), pulmonary hypertension (PH), or other diagnoses. The referral period was grouped into 3 time periods (2012-2017, 2018-2021, and 2022-2025) to account for changes in testing practices over time.

Clinical characteristics included assessment of outcomes, COT, and treating physician. Transplant assessment outcome was categorized as screened, rejected due to modifiable contraindications (mod CI) (e.g., physical deconditioning or nicotine other substance abuse), rejected due to non-modifiable contraindications (non-mod CI) (e.g., active malignancies or heart failure), patient deemed too early for LTx, LTx declined by patient, or other reasons (e.g., lost to follow-up/deceased).

### Outcomes

Self-reported nicotine use was extracted from electronic medical records. Biochemical verification of nicotine exposure was performed using urinary cotinine measurements, analyzed by immunoassay (ARCHITECT, Abbott Diagnostics). Cotinine concentrations <100 μg/L were classified as negative; levels of 100 to 499 μg/L were considered positive and consistent with passive nicotine exposure, although recent or intermittent active use could not be excluded on this basis alone, whereas levels ≥500 μg/L were interpreted as positive and indicative of active nicotine use. Smoking resumption after LTx was defined as a urinary cotinine concentration ≥500 μg/L or ≥100 μg/L in combination with self-reported active smoking during routine post-transplant follow-up. In patients with a positive COT at referral, smoking cessation was assessed during a 6-month period in which patients were required to provide urine samples every 2 weeks via their general practitioner or referring physician. If all samples were negative for nicotine use, patients were re-evaluated for transplant evaluation.

### Statistical analysis

Data are presented as mean with standard deviation, median with interquartile range, or as number with percentage. Group comparisons for continuous variables were performed with the Mann-Whitney *U* test or Kruskal-Wallis test, and for categorical variables with the Chi-square test or Fisher’s exact tests. To identify factors associated with cotinine test ordering and subsequently a positive test results, we performed univariable logistic regression and subsequently multivariable logistic regression for both outcomes with significant variables (*p* < 0.1). Factors included age (years), sex (male/female), referral period (2012-2017, 2018-2021, or 2022-2025), underlying disease (COPD, ILD, CF/BE, PH, or other), former smoker (yes/no), pack-years (years), time since nicotine cessation (years), the presence of smoking household members (yes/no), screening status (screened, rejected with mod CI, rejected with non-mod CI, deemed too early for LTx, LTx declined by patient, or other), and treating physician (physician 1-4, or other). In the multivariable analyses, pulmonary hypertension was grouped with “other” because of small numbers. Due to multicollinearity between smoking-related variables, former smoking status was preferred for inclusion in the multivariable model for test ordering over pack-years and time since nicotine cessation. Likewise, we explored factors associated with positive nicotine exposure among tested patients, with underlying disease categorized as COPD vs non-COPD diagnoses. As nearly all patients with a positive test were former smokers (109/110), time since nicotine cessation was included instead of former smoking status in this model. Because assessment outcome may be both a reason for and a consequence of testing (e.g., ongoing nicotine use constitutes a modifiable contraindication), associations with assessment outcome were not interpreted as temporal predictors. As a sensitivity analysis, a mixed-effects logistic regression model with a random intercept for physician was fitted to account for clustering of certain patient categories within physicians and unmeasured differences in individual testing behavior. To estimate the positivity rate in the total referral cohort, a sensitivity analysis was performed using best-case (all non-tested patients negative) and worst-case (all non-tested patients positive) assumptions, and by applying the positivity rates observed among tested patients, stratified by smoking history, to non-tested patients. Time to smoking resumption after transplantation was analyzed using cumulative incidence functions (Aalen-Johansen estimator), with death without smoking resumption treated as a competing event. Patients alive and without smoking resumption at the end of follow-up (31 December 2025) were censored. A *p*-value <0.05 was considered statistically significant. Statistical analyses were performed with R statistical software version 4.5.2.

## Results

From January 1, 2012, to December 31, 2025, 1,326 patients were referred for LTx work-up in our center. The median age was 59 (52-63) years, and 52% was female (Table 1). The underlying diseases included COPD (*n* = 737, 56%), ILD (*n* = 475, 36%), CF/BE (*n* = 59, 4.4%), PH (*n* = 31, 2.3%), and others (*n* = 24, 1.8%).

**Table 1:** Demographics and Clinical Characteristics of Patients Referred for Lung Transplant Evaluation

	*N*	All Patients *N* = 1,326	Non-tested *n* = 752 (57%)	Tested *n* = 574 (43%)	*p*-value
*Demographics*					
Age, years	1,326	59 [52,63]	58 [51,63]	60 [55,64]	<0.001
Sex, female	1,326	685 (52%)	384 (51%)	301 (52%)	0.7
Referral period*	1,326				<0.001
2012-2017		356 (27%)	319 (42%)	37 (6.4%)	
2018-2021		484 (37%)	206 (27%)	278 (48%)	
2022-2025		486 (37%)	227 (30%)	259 (45%)	
*Underlying disease*	1,326				<0.001
COPD		737 (56%)	338 (45%)	399 (70%)	
ILD		475 (36%)	317 (42%)	158 (28%)	
CF/BE		59 (4.4%)	49 (6.5%)	10 (1.7%)	
PH		31 (2.3%)	28 (3.7%)	3 (0.5%)	
Other		24 (1.8%)	20 (2.7%)	4 (0.7%)	
Former smoker	1,326	1065 (80%)	544 (72%)	521 (91%)	<0.001
Pack-years	758	30 [15,40]	25 [14,36]	30 [18,40]	<0.001
Years since nicotine cessation	983	6 [2,12]	6 [2,15]	5 [2,11]	0.008
Smoking housemates	1,326	123 (9.3%)	41 (5.5%)	82 (14%)	<0.001
*Clinical characteristics*					
*Assessment outcome*	1,326				<0.001
Screened		584 (44%)	322 (43%)	262 (46%)	
Non-modifiable CI		262 (20%)	167 (22%)	95 (17%)	
Modifiable CI		120 (9.0%)	42 (5.6%)	78 (14%)	
Too early for LTx		218 (16%)	138 (18%)	80 (14%)	
LTx declined by patient		95 (7.2%)	59 (7.8%)	36 (6.3%)	
LTFU/Other		47 (3.5%)	24 (3.2%)	23 (4.0%)	
Physician*	1,326				<0.001
Physician 1		301	135 (45%)	166 (55%)	
Physician 2		263	113 (43%)	150 (57%)	
Physician 3		213	109 (51%)	104 (49%)	
Physician 4		137	63 (46%)	74 (54%)	
Other		412	332 (81%)	80 (19%)	

Data are presented as median (interquartile range) or *n* (%). *P*-values are based on Mann-Whitney *U* test for continuous variables and Chi-square or Fisher’s exact tests for categorical variables. * For referral period and physician, row percentages are presented, based on the total number of patients per referral period or per physician (e.g., 135 of 301 patients: 45%).

Abbreviations: BE, non-CF bronchiectasis; CF, cystic fibrosis; CI, contraindication; COPD, chronic obstructive pulmonary disease; ILD, interstitial lung disease; LTFU, lost to follow-up; LTx, lung transplantation; PH, pulmonary hypertension.

### Characteristics of COT tested vs COT non-tested group

Of the 1,326 patients referred and assessed, 574 (43%) patients underwent COT (Figure 1). Tested patients were significantly older (60 vs 58 years, *p* < 0.001) and more often referred in recent years, with testing increasing over time (10% in 2012-2017, 57% in 2018-2021 and 53% in 2022-2025, *p* < 0.001). The tested group comprised more patients with COPD (70% vs 45%) and fewer with ILD (28% vs 42%, *p* < 0.001), had more often a history of smoking (91% vs 72%, *p* < 0.001), higher pack-year exposure (30 vs 25 pack-years, *p* < 0.001), shorter duration of nicotine abstinence (5.0 vs 6.0 years, *p* = 0.008), and more frequently lived with smoking housemates (14% vs 5.5%, *p* < 0.001). In addition, they were more often classified as having modifiable contraindications (14% vs 5.6%).

**Figure 1:**
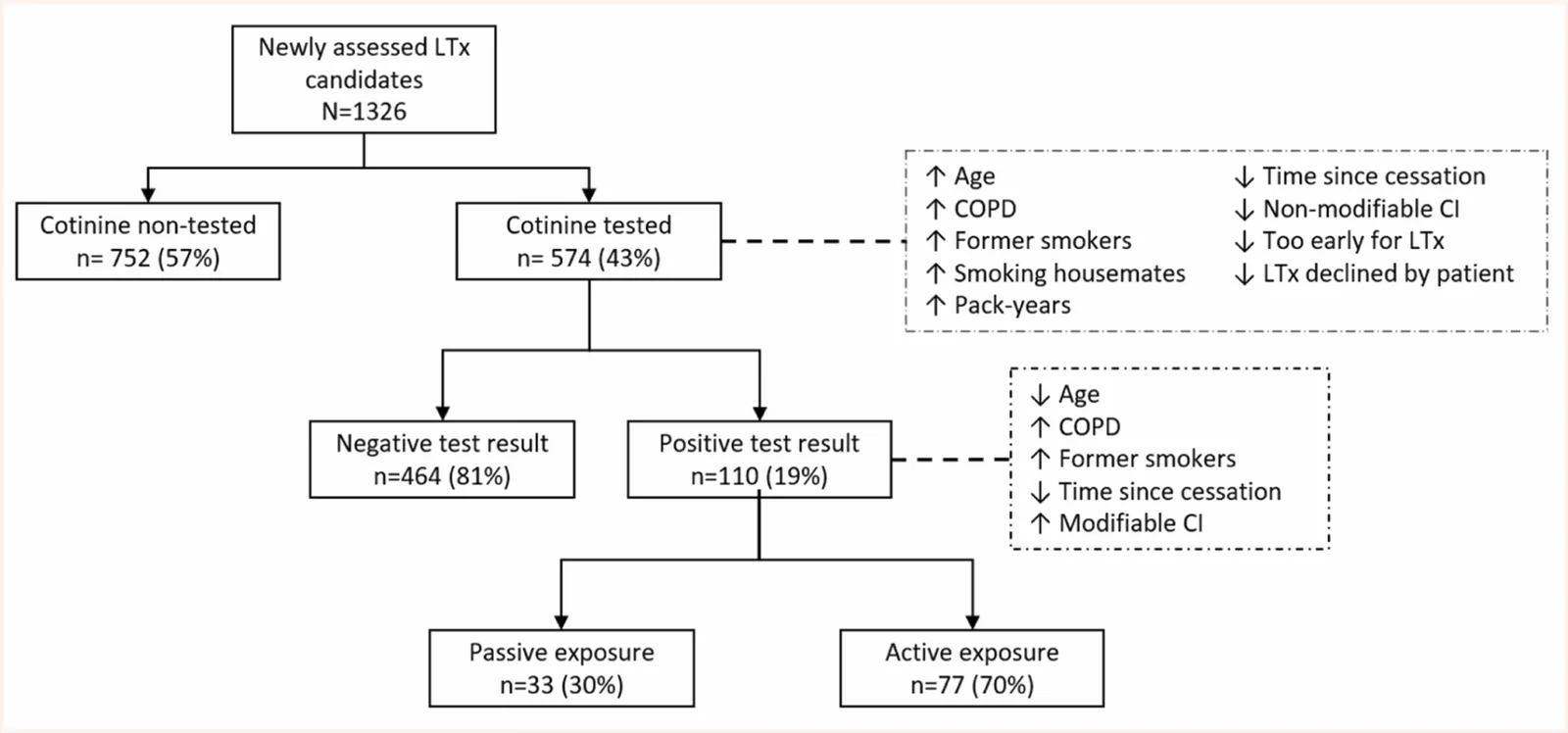
Flowchart of study cohort. Dashed squares represent characteristics of the group. ↑ indicates higher prevalence or longer time, ↓ indicates lower prevalence or shorter time. LTx, lung transplantation; COPD, chronic obstructive pulmonary disease; ILD, interstitial lung disease; CI, contraindication.

The proportion of patients tested ranged from 49% to 57% among the 4 currently active physicians, compared with 19% among other physicians (*p* < 0.001).

### Factors associated with cotinine test ordering

Results of univariable logistic regression analyses are presented in SFigure 1. In multivariable analysis, the more recent referral period remained the strongest independent predictor of COT (Figure 2). Compared with patients with COPD, those with ILD and those classified under other diagnoses were significantly less likely to be tested. Former smoking status remained positively associated with COT, as was the presence of smoking household members. Patients rejected due to non-modifiable contraindications, deemed too early for LTx, or who declined LTx were less likely to undergo testing than patients who proceeded to full transplant evaluation. After adjustment for patient characteristics, physician 3 and other physicians were less likely to order COT than physician 1 (Figure 2), suggesting that the similar crude testing rates among the currently active physicians partly reflected differences in case mix.

**Figure 2:**
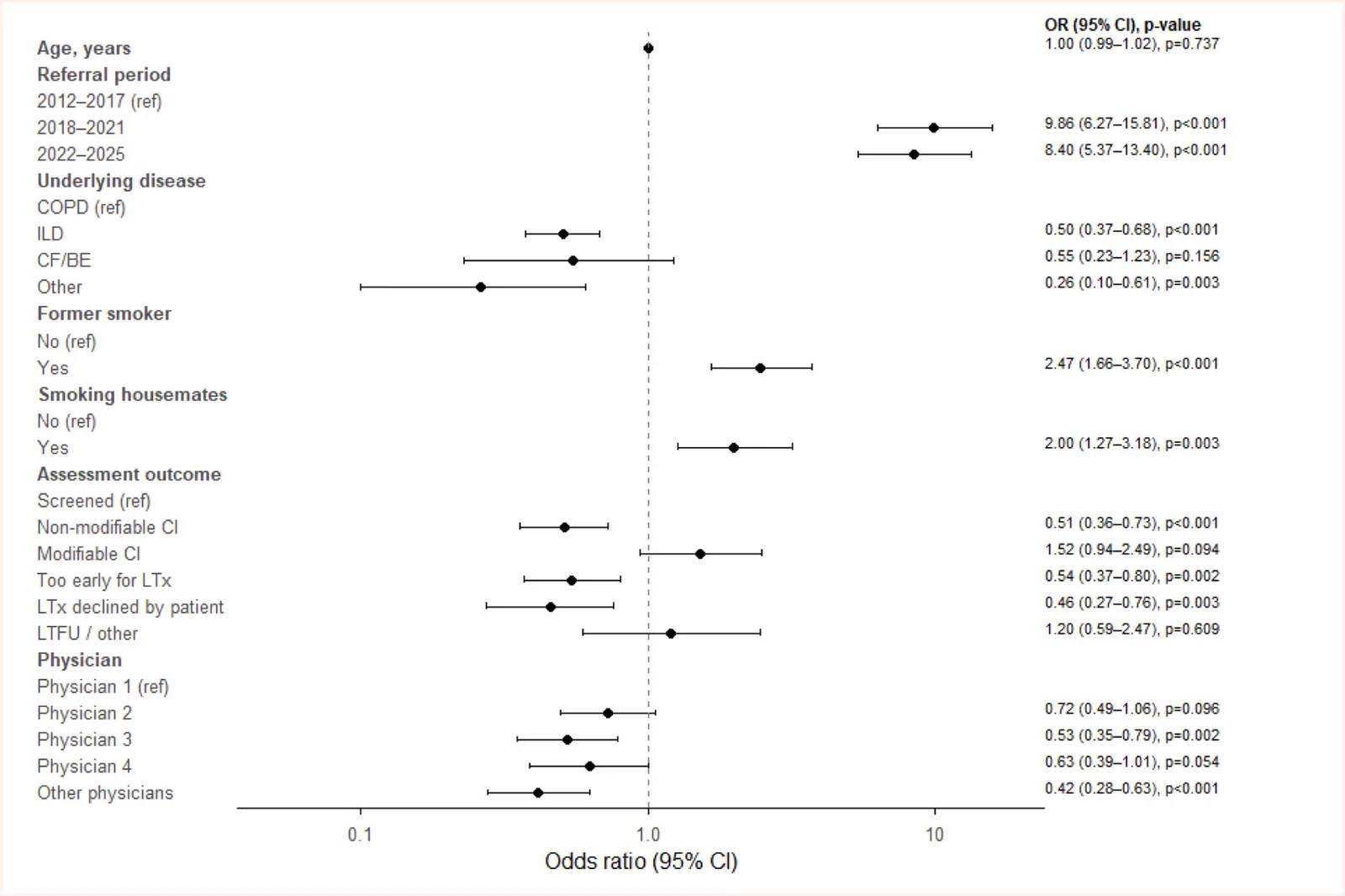
Forest plot showing adjusted odds ratios with 95% confidence interval from multivariable logistic regression analysis of cotinine test ordering (yes/no) at lung transplantation referral. COPD, chronic obstructive pulmonary disease; ILD, interstitial lung disease; CF, cystic fibrosis; BE, non-CF bronchiectasis; CI, contraindication; LTx, lung transplantation; LTFU, lost to follow-up.

Sensitivity analyses yielded consistent findings, indicating that the findings were robust and not driven by variation in testing behavior between individual physicians (SFigure 2).

### COT results

Among the 574 (43% of total referrals) patients tested for cotinine, 464 (81%) had negative levels and 110 (19%) had a positive result; of these, 77 (13% of tested) were indicative of active nicotine use and 33 (6% of tested) suggested passive exposure (Table 2). In a sensitivity analysis, the estimated positivity rate in the total referral cohort ranged from 8.3% (best case, assuming all non-tested patients were negative) to 65% (worst case, assuming all non-tested patients were positive). Applying the positivity rates observed among tested former smokers (20.9%) and never-smokers (1.9%) to non-tested patients resulted in an estimated positivity rate of 17.2% in the total referral cohort. Patients with active exposure were younger than those with passive exposure and negative tests (56 vs 59 and 61 years, *p* < 0.001, respectively). Underlying disease distribution differed significantly (*p* < 0.001), with COPD most prevalent in the active group (92% vs 82% passive and 65% negative) and ILD least frequent (7.8% vs 15% and 32%, respectively). All active users were former smokers, and the duration of nicotine cessation was shorter in the passive and active groups than in the negative group (2 and 3 years vs 6 years, *p* < 0.001, respectively). Notably, patients with a positive test result were less likely to proceed to full LTx screening than those with a negative result (19% vs 52%). Of these patients, 14/110 were eventually transplanted after completing the controlled cessation program described in the Methods section, of whom 2 (14%) resumed smoking after LTx.

**Table 2:** Demographics and Clinical Characteristics by COT Result: Negative, Positive Passive, and Positive Active

	Negative *n* = 464 (81%)	Positive passive *n* = 33 (6%)	Positive active *n* = 77 (13%)	*p*-value
*Demographics*				
Age, years	61 [55,64]	59 [55,63]	56 [53,60]	<0.001
Sex, female	238 (51%)	19 (58%)	44 (57%)	0.5
Referral period				0.008
2012-2017	32 (6.9%)	3 (9.1%)	2 (2.6%)	
2018-2021	216 (47%)	11 (33%)	51 (66%)	
2022-2025	216 (47%)	19 (58%)	24 (31%)	
Underlying disease				<0.001
COPD	301 (65%)	27 (82%)	71 (92%)	
ILD	147 (32%)	5 (15%)	6 (7.8%)	
CF/BE	10 (2.2%)	0 (0%)	0 (0%)	
PH	2 (0.4%)	1 (3.0%)	0 (0%)	
Other	4 (0.9%)	0 (0%)	0 (0%)	
Former smoker	412 (89%)	32 (97%)	77 (100%)	0.021
Pack-years	30 [16,40]	30 [20,40]	35 [25,41]	0.056
Years since nicotine cessation	6 [2,12]	2 [1,7]	3 [1,7]	<0.001
Smoking housemates	59 (13%)	9 (27%)	14 (18%)	0.095
*Clinical characteristics*				
Assessment outcome				<0.001
Screened	241 (52%)	8 (24%)	13 (17%)	
Non-modifiable CI	80 (17%)	5 (15%)	10 (13%)	
Modifiable CI	24 (5.2%)	10 (30%)	44 (57%)	
Too early for LTx	71 (15%)	3 (9.1%)	6 (7.8%)	
LTx declined by patient	29 (6.3%)	4 (12%)	3 (3.9%)	
LTFU/Other	19 (4.1%)	3 (9.1%)	1 (1.3%)	
Physician *				0.10
Physician 1	129 (77%)	10 (6.0%)	27 (16%)	
Physician 2	117 (78%)	6 (4.0%)	27 (18%)	
Physician 3	83 (80%)	10 (9.6%)	11 (11%)	
Physician 4	63 (85%)	4 (5.4%)	7 (9.4%)	
Other	72 (90%)	3 (3.8%)	5 (6.3%)	

Continuous variables are presented as median (IQR). Categorical variables are presented as *n* (%). *P*-values are based on Kruskal–Wallis tests for continuous variables and Chi-square or Fisher’s exact tests for categorical variables. * For physician, row percentages are presented, based on the total number of tested patients per referral period or per physician (e.g., 129 of 166 patients: 78%).

BE, non-CF bronchiectasis; CF, cystic fibrosis; CI, contraindication; COPD, chronic obstructive pulmonary disease; ILD, interstitial lung disease; LTFU, lost to follow-up; LTx, lung transplantation; PH, pulmonary hypertension.

Results of univariable analysis are presented in SFigure 3. Younger age, shorter time since nicotine cessation, and presence of modifiable contraindications remained independently associated with cotinine positivity in the multivariable analysis (Figure 3). The association with modifiable contraindications largely reflects that ongoing nicotine use itself constitutes a modifiable contraindication. Patients with active cotinine exposure were significantly younger than those with passive exposure (median difference 3 years, *p* = 0.01); other variables were comparable between actively and passively exposed groups.

**Figure 3:**
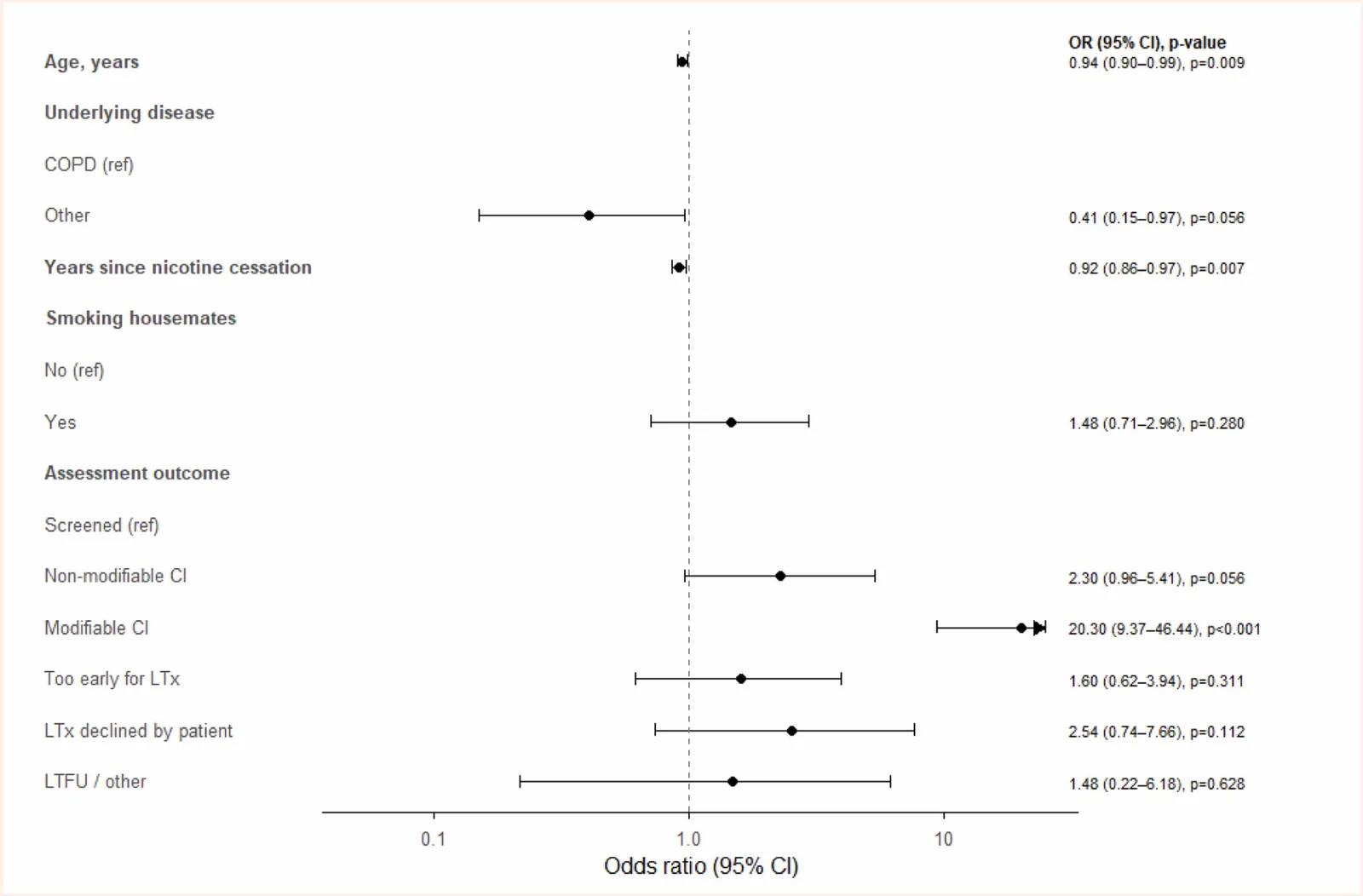
Forest plot showing adjusted odds ratios with 95% confidence interval from multivariable logistic regression analysis of cotinine test result (positive/negative) at lung transplantation referral. COPD, chronic obstructive pulmonary disease; CI, contraindication; LTx, lung transplantation; LTFU, lost to follow-up.

### Post-LTx

Between 2012 and 2025, 305 patients underwent LTx, of whom 12 (3.9%) resumed smoking. This corresponded to a cumulative incidence of 1.0% at 1 year and 4.2% at 5 years post-transplantation (Figure 4). Median time to smoking resumption was 1.2 years after LTx, with the earliest resumption detected at first routine evaluation (i.e., 6 months after discharge) and all others within 3 years.

**Figure 4:**
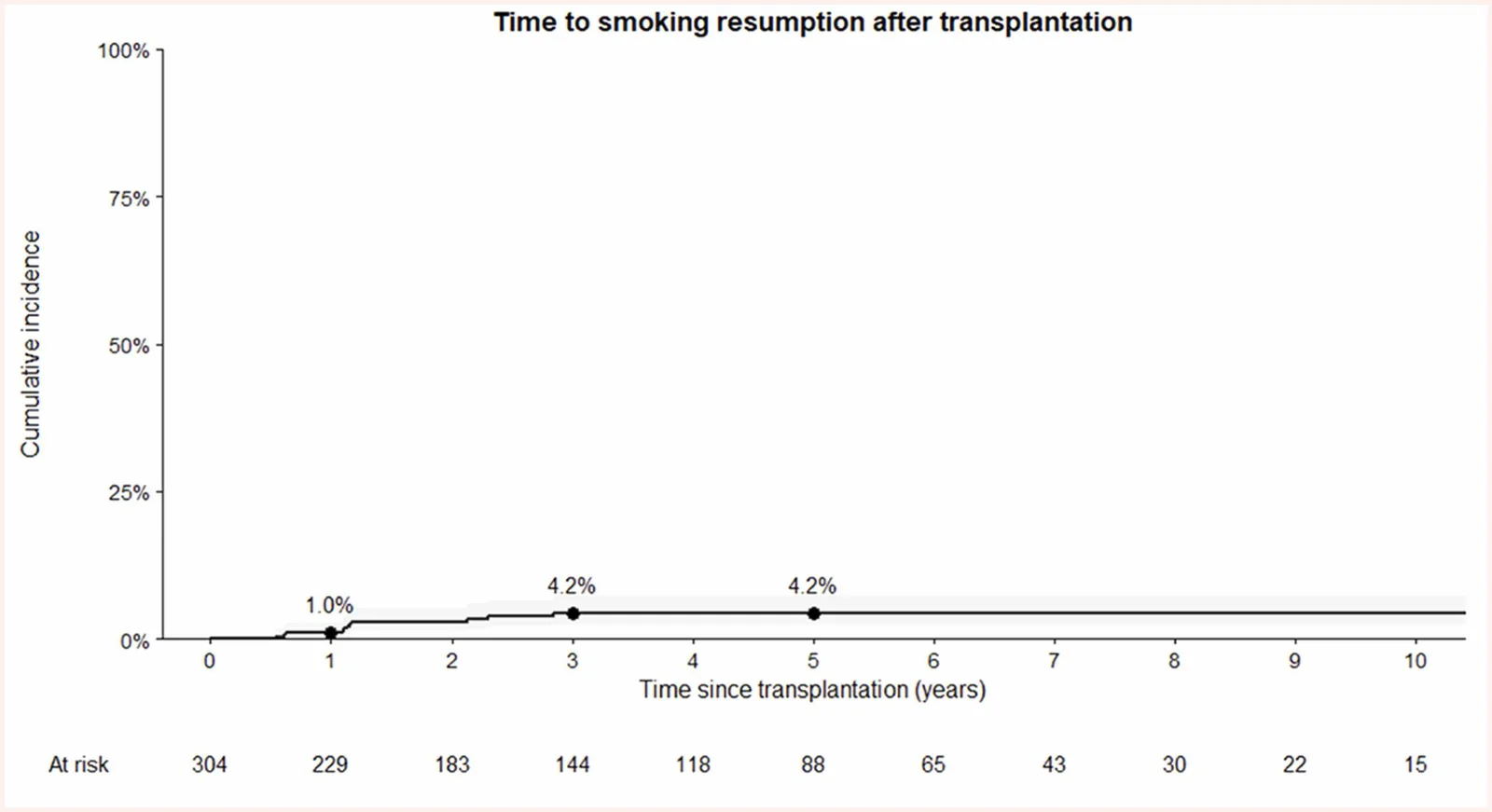
Cumulative incidence of smoking resumption after lung transplantation, estimated with the Aalen–Johansen method. Points and percentages indicate the cumulative incidence at 1, 3, and 5 years. The shaded area represents the 95% confidence interval. The number of patients at risk at each time point is shown below the figure.

Among patients who resumed smoking, 67% were male, all had a history of smoking, 2 had ILD and all others COPD, one (8%) reported smoking household members, and 50% underwent COT at the initial visit. Two of the tested patients had a positive test at referral and completed the controlled cessation program prior to acceptance for full evaluation. Median pack-years was 29 (19-38) and median time since nicotine cessation was 2 (0.7-3.0) years.

## Discussion

In this retrospective cohort study of LTx candidates, we evaluated how routinely and consistently COT is applied during initial LTx referral workup and identified factors associated with test ordering. In addition, we assessed the prevalence and correlates of a positive cotinine test at evaluation and smoking resumption after LTx.

We observed that COT was performed in fewer than half of all referred patients and was strongly influenced by patient-, clinical-, and physician-related factors. Among tested patients, a clinically relevant prevalence (19%) of passive and active nicotine exposure was identified at referral, with a considerable proportion of active users despite self-reported abstinence. As testing was performed at the discretion of the treating physician, this positivity rate reflects the yield of COT as applied in daily clinical practice rather than the prevalence in all referred patients, which we estimated at 17.2% in a sensitivity analysis.

We acknowledge that a positive COT does not distinguish between conventional cigarette use, e-cigarette use, and nicotine replacement therapy. However, as any form of nicotine dependence is considered a risk factor for relapse to tobacco smoking, all are treated equally in our center and regarded as a contraindication to transplantation.

The characteristics associated with cotinine positivity in our cohort are consistent with previously described predictors of cotinine positivity and established risk profiles for relapse post-transplantation.^2^, ^3^, ^4^, ^5^, ^10^, ^11^, ^13^ Importantly, these patients were also less likely to progress to full screening and undergo LTx, underscoring the clinical consequences of concealed nicotine use. Physician-level variability in COT practices influenced whether nicotine exposure was identified at all, highlighting an implementation gap that has received limited attention in prior studies.^13^

These findings are in line with a recent smaller study by Zuazaga-Fuentes et al, which similarly evaluated COT during LTx referral workup.^11^ That study reported a higher testing rate (76%) and a comparable positivity rate (14.6%), further supporting the relevance of biochemical verification at referral. However, it did not address how COT is implemented in clinical practice, including which factors determine test ordering, inter-physician variability, associations with ongoing nicotine exposure, or the impact of testing on subsequent transplant evaluation and post-transplant outcomes. Together, these findings form a strong base to emphasize the importance of biochemical verification of nicotine abstinence early after referral for LTx.^9^

Although testing increased over time, reflecting gradual uptake, it was not applied universally. This selective approach appears partly intuitive: candidates with non-modifiable contraindications, those who declined LTx, and never-smokers with non-smoking-related diagnoses, such as CF or selected ILD cases, were less likely to be tested, which may be clinically justified. In contrast, patients deemed too early for LTx were also less frequently tested, representing a potential missed opportunity, as early referral allows for smoking cessation support and clinical optimization that might even avert the need for transplantation. As expected, testing was more commonly performed in patients perceived to be at higher risk, including those with a smoking history, COPD, smoking household exposure, or modifiable contraindications. This selective application aligns with earlier observations that smoking assessment in transplant populations often depends on patient characteristics and individual clinician judgment rather than standardized protocols, leading to inconsistent assessment of nicotine abstinence.^10^, ^11^, ^13^ While this risk-based approach may appear pragmatic, it risks missing active nicotine use in patients outside traditional high-risk profiles, which were also represented in this cohort.

Our findings further reinforce the limitations of relying on self-reported smoking history alone and support biochemical verification of nicotine abstinence.^10^, ^11^, ^12^ Given the well-established association between smoking and adverse post-transplant outcomes as well as misallocation of donor organs, accurate identification of nicotine use prior to listing remains essential.^2^, ^5^, ^6^, ^7^ Because of the overrepresentation of high-risk patients in the COT tested group, it is reasonable to assume that expanding COT as a standard to all referred patients will lead to a lower positivity rate. Universal testing may reduce efficiency and increase unnecessary testing in patients with a low pretest probability of nicotine use.

Therefore, we present a conceptual framework requiring future validation for implementation of a structured, risk-based urinary COT strategy at the time of LTx referral to reduce physician-dependent variability, maintaining a rational rather than a one-size-fits-all approach (Figure 5). Based on our findings and experience, a more selective, probability-based approach to nicotine testing, rather than universal testing at the first visit, may be considered. Cotinine testing may be omitted in patients with non-modifiable contraindications and in those who decline transplantation, as the result will not influence clinical management. In never-smokers and patients with long-term abstinence without household smoking exposure, the pre-test probability of nicotine exposure is low, and testing may be deferred; in our cohort, only 1 of 53 tested never-smokers had a positive result, consistent with passive exposure. In all remaining candidates, including those deemed too early for transplantation but presenting with a risk profile (history of smoking, COPD, recent nicotine cessation, or smoking household exposure), we suggest testing to enable timely identification of and intervention for concealed nicotine use. As the absence of these risk factors does not fully exclude ongoing nicotine use, the safety of deferring testing in low-risk patients requires prospective validation. This approach would further standardize practice.

**Figure 5:**
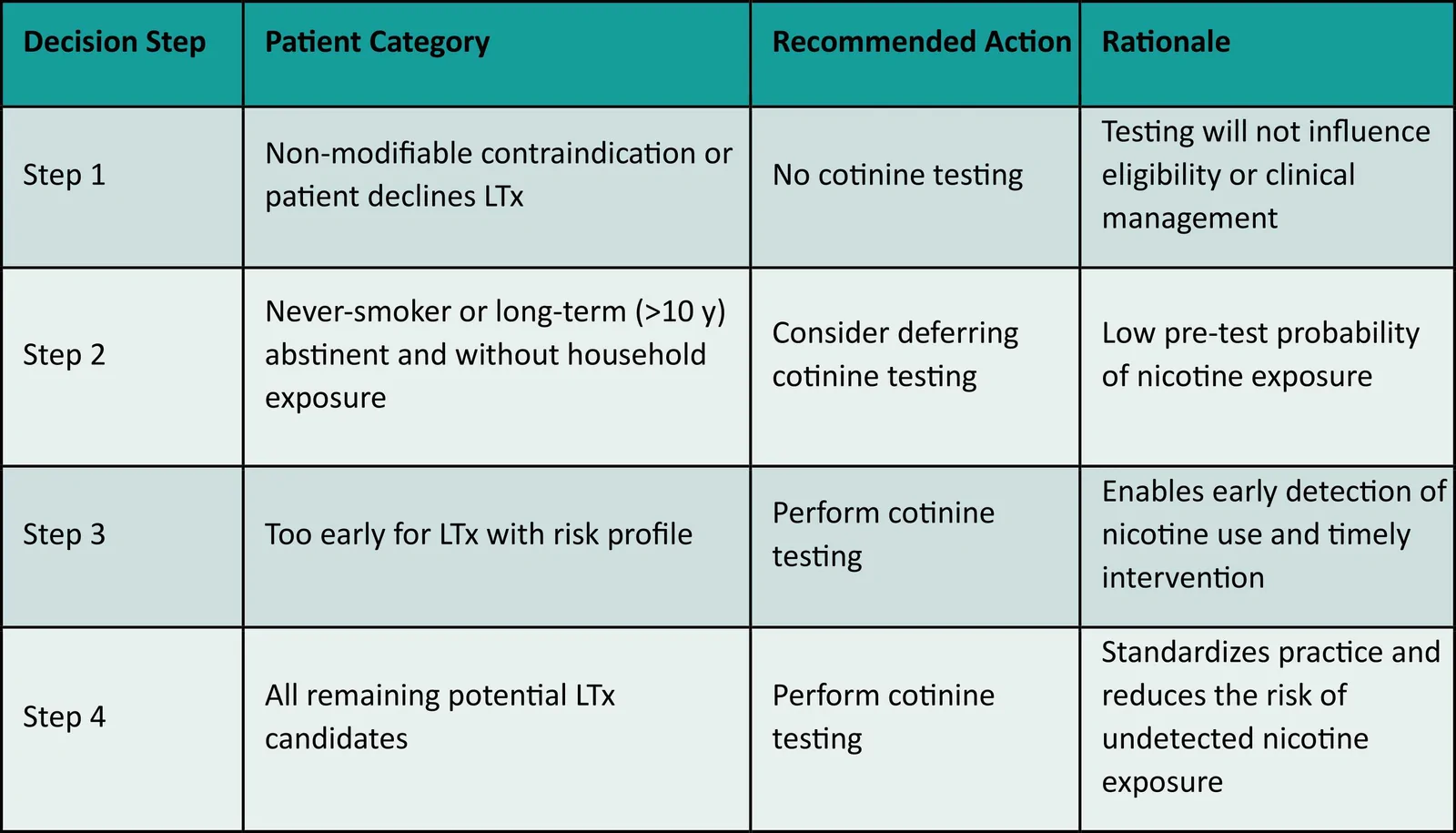
Proposed risk‑based conceptual framework for urinary cotinine testing during lung transplant candidate evaluation, which requires prospective validation before implementation in clinical practice.

In a simulation of this framework in our cohort, 44.2% of all referred patients would remain eligible for COT after exclusion of directly unsuitable candidates (i.e., non-modifiable contraindications or LTx declined by the patient) and individuals without meaningful smoking exposure (i.e., never-smokers or those who had stopped smoking more than 10 years before evaluation). This compares with a testing rate of 76% in another cohort reporting a similar positivity rate (14.6%), predominantly among former smokers.^11^ This suggests that a targeted, decision-rule-based strategy as proposed here, instead of a one-size-fits-all approach, may render implementation more efficient and resource-conscious. However, as cotinine results are unknown in non-tested patients, whether this approach maintains detection of clinically relevant nicotine use requires prospective validation. Post-LTx, the cumulative incidence of active smoking relapse was 4.2% at 5 years, substantially lower than the 12% to 19% reported previously.^2^, ^3^, ^4^ We hypothesize that this may reflect stricter candidate selection and improved pre-transplant screening, with COT facilitating early detection of concealed nicotine use before transplantation, preventing high-risk candidates from proceeding to transplantation. In addition, awareness of biochemical monitoring may itself discourage smoking and contribute to lower post-transplant relapse rates. Alternatively, differences in study period and patient population compared with earlier reports may contribute to this difference. As this study was not designed to assess the effect of COT on smoking relapse, these hypotheses warrant further evaluation.

This study has several strengths, including a large cohort, detailed demographic and clinical characterization, and integration of physician-level factors, allowing a nuanced assessment of real-world practice of COT implementation. Nonetheless, several limitations were present. Because only tested patients could be classified into cotinine exposure categories and high-risk patients were overrepresented in the tested group, the observed positivity rate likely somewhat overestimates the true prevalence of nicotine exposure in the overall referral cohort, as also suggested by our sensitivity analysis. In addition, the number of smoking resumption events after LTx was limited (*n* = 12), and these estimates should therefore be regarded as descriptive. While immunoassay-based testing is not the most specific test available, the clinical relevance of this difference is debatable in the context of our screening protocol. The primary objective is not to determine the exact cotinine concentration but rather to identify a positive test result that can be discussed with the patient and used to guide clinical decision-making. Finally, the single center setting may limit generalizability, although the observed variability in testing practices likely reflects challenges common to other transplant centers.

In conclusion, COT during lung transplant candidate evaluation is applied variably, based on patient characteristics and clinician practice rather than standardized criteria. A substantial proportion of tested candidates demonstrated passive or active nicotine exposure at referral, often despite self-reported abstinence. Notably, a low rate of smoking resumption post-LTx was observed. These findings support further evaluation of a more structured, risk-based cotinine testing strategy in order to accurately identify ongoing nicotine use early, select the best candidates for LTx while precluding inappropriate organ allocation of scarce donor organs, and reach the best LTx outcomes.

## Ethics Approval

The Medical Ethics Committee of the Erasmus Medical Center approved the study (MEC-2025–0632).

## Data Availability

All data generated or analyzed during this study are included in this article. Further inquiries can be directed to the corresponding author.

## Conflicts of Interest statement

The authors declare that they have no known competing financial interests or personal relationships that could have appeared to influence the work reported in this paper.

## Footnotes

Appendix A Supplementary data associated with this article can be found in the online version at doi:10.1016/j.jhlto.2026.100709.
