# Supplementary material for: Beyond trust: Implementation of urinary cotinine testing in lung transplant candidate evaluation
Source: JHLT Open. 2026 Oct 16;15:100709. doi: 10.1016/j.jhlto.2026.100709 (PMC13756532; doi:10.1016/j.jhlto.2026.100709)
Supplement: Supplementary file 1 [file mmc1.docx]

#### Supplemental material

**Supplementary Figure 1**. Forest plot showing odds ratios with 95% confidence interval from univariable logistic regression analysis of cotinine test ordering (yes/no) at lung transplantation referral. COPD, chronic obstructive pulmonary disease; ILD, interstitial lung disease; CF, cystic fibrosis; BE, non-CF bronchiectasis; CI, contraindication; LTx, lung transplantation; LTFU, lost to follow-up.

**Supplementary Figure 2.** Forest plot showing results of the sensitivity analyses presenting adjusted odds ratios from multivariable fixed- and random-effects logistic regression models for cotinine test ordering (yes/no) at lung transplantation referral. COPD, chronic obstructive pulmonary disease; ILD, interstitial lung disease; PH, pulmonary hypertension; CF, cystic fibrosis; BE, non-CF bronchiectasis; CI, contraindication; LTx, lung transplantation; LTFU, lost to follow-up.

 **Supplementary Figure 3.** Forest plot showing odds ratios with 95% confidence interval from univariable logistic regression analysis of cotinine test result (positive/negative) at lung transplantation referral. COPD, chronic obstructive pulmonary disease; CI, contraindication; LTx, lung transplantation; LTFU, lost to follow-up.
